# Optogenetics: implications for Alzheimer’s disease research and therapy

**DOI:** 10.1186/s13041-022-00905-y

**Published:** 2022-02-23

**Authors:** Parsa Mirzayi, Parnian Shobeiri, Amirali Kalantari, George Perry, Nima Rezaei

**Affiliations:** 1 School of Medicine, Tehran University of Medical Sciences (TUMS), Children’s Medical Center Hospital, Dr. Qarib St., Keshavarz Blvd, 14194 Tehran, Iran; 2 Network of Immunity in Infection, Malignancy and Autoimmunity (NIIMA), Universal Scientific Education and Research Network (USERN), Tehran, Iran; 3 Non–Communicable Diseases Research Center, Endocrinology and Metabolism Population Sciences Institute, Tehran University of Medical Sciences, Tehran, Iran; 4 Research Center for Immunodeficiencies, Pediatrics Center of Excellence, Children’s Medical Center, Tehran University of Medical Sciences, Tehran, Iran; 5 Department of Biology and Neurosciences Institute, University of Texas at San Antonio (UTSA), San Antonio, TX USA; 6 Department of Immunology, School of Medicine, Tehran University of Medical Sciences, Tehran, Iran; 7 Research Center for Immunodeficiencies, Children’s Medical Center, Dr. Gharib St, Keshavarz Blvd, Tehran, Iran

**Keywords:** Alzheimer’s disease, Optogenetics, Neural circuits, Neurodegeneration, Synapse, Molecular pathways, Memory

## Abstract

Alzheimer’s disease (AD), a critical neurodegenerative condition, has a wide range of effects on brain activity. Synaptic plasticity and neuronal circuits are the most vulnerable in Alzheimer’s disease, but the exact mechanism is unknown. Incorporating optogenetics into the study of AD has resulted in a significant leap in this field during the last decades, kicking off a revolution in our knowledge of the networks that underpin cognitive functions. In Alzheimer's disease, optogenetics can help to reduce and reverse neural circuit and memory impairments. Here we review how optogenetically driven methods have helped expand our knowledge of Alzheimer's disease, and how optogenetic interventions hint at a future translation into therapeutic possibilities for further utilization in clinical settings. In conclusion, neuroscience has witnessed one of its largest revolutions following the introduction of optogenetics into the field.

## Introduction

“A peculiar severe disease process of the cerebral cortex” are the words used by A. Alzheimer to describe his patient's severe memory loss, personality changes, and sleep disturbance in 1906. This condition is now known as Alzheimer’s disease (AD), the world's leading cause of dementia, the most common neurodegenerative disease, and the 5th overall cause of mortality affecting ~ 44 million people globally [1]. At the same time, even though several theories for AD etiology have emerged throughout the past century, its etiology and pathology remain relatively unclear, and no successful treatment exists [2].

Francis Crick once suggested that the main challenge facing neuroscience is to control one cell type in the brain while leaving others untouched [3]. The remarkable specificity of optogenetic techniques, first introduced in 2005 by a pair of researchers [4, 5], has allowed scientists to address this issue. Researches can now target very specific populations of cells within (or sometimes across) tissues and observe neural networks in a much higher resolution [6, 7], helping to better dissect the pathology behind several neurologic conditions [8]. Optogenetics is using genetically modified cells expressing proteins that respond to light [9]. This method has been an important milestone in neuroscience, aiding researchers in discovering and modulating neural circuits that govern different complex functions of behavior and cognition [10–13]. Optogenetic findings can determine dependencies and infer causality, which is necessary for establishing circuit-centric therapeutics, thus overcoming the limitations of most other approaches [14].

Here we review recent breakthroughs in AD research and discuss what the future clinical horizon looks like. Although optogenetics has had broad uses in neuroscience studies, we have only chosen studies that have been used on AD models or have clear implications for understanding or treating AD.

## Introduction to optogenetic methods

### Opsins

Rhodopsins (named for their light-responsive molecule opsin), first reported in 1971 [15], are light-sensitive proteins that are expressed in all organisms [16]. These transmembrane proteins respond to different wavelengths of electromagnetic light, thus initiating ion exchanges that alter the plasma membrane potential. The central idea behind optogenetics is fine-tuning these proteins to fit into specific subpopulations of cells and then inducing said membrane potential changes using light [17].

Optogenetics studies commonly use microbial opsins because of their efficient kinetics and that they are relatively simpler to engineer [18]. These including ion pumps (such as bacteriorhodopsins and halorhodopsins) and ion channels (such as channelrhodopsins) [19]. Opsins have been rigorously engineered to offer a complex toolbox for neuronal excitation or inhibition, as well as control of intracellular signaling [20, 21], rendering optogenetics a powerful method to modulate molecular events in a targeted manner.

### Gene vehicles

Opsin-encoding genes must be delivered into the desired cell population, and various delivery methods exist for this purpose. Lenti- and adeno-associated viral vectors are most commonly used to transfer the opsin gene into the desired cell population [13, 17]. Viruses allow the simultaneous induction of activation or inhibition in neural cells of the same class, even if scattered throughout the tissue and offer an extraordinary efficacy in invading living cells [4]. Non-viral vectors are also being explored; these vehicles are safer, cost-effective, and relatively easier to use; but have lower delivery efficacy and expression rates [22].

### Light delivery

The main methods of introducing light into the tissue are (1) distant light sources that require infiltration of the tissue, typically through an optical fiber, and (2) in-site light sources (such as LEDs), which can either be implanted [23] or directly target the cortical surface [24, 25]. LEDs and lasers are widely used due to their simplicity, effectiveness, and cost. Their disadvantage is having a narrow spectral bandwidth requiring two different devices to activate two spectrally different opsins independently [26].

Optical fibers can potentially be connected to any light source as long as their power is controlled. This control is necessary so that the tissue is exposed to as little light as possible to minimize heat and light damage, but also sufficient light to activate the opsins. Standard white light sources work in this setting, but given that light should be band-passed to reduce the power and opsins have different sensitivities to wavelengths, the right filter should be applied [27].

## Role of optogenetics in revealing Alzheimer’s disease pathogenesis

There is no established consensus as to what clinical findings correspond with AD. However, AD is famously characterized by the presence of amyloid-beta (Aβ) plaques and neurofibrillary tau tangles [28]. Researchers once suggested the deposition of amyloid-beta plaques in different regions of the brain as the primary molecular basis for pathogenesis [29, 30], which, following inconsistencies [31], was modified to be smaller, more soluble amyloid oligomer assemblies rather than plaques [32, 33]. Now it is thought that AD begins with minute alterations of hippocampal synaptic function induced by diffusible oligomeric assemblies of Aβ [34], specifically AβO_1–42_ [35]. The rest of the disease process, including the formation of tau protein tangles, results from a production-clearance imbalance of these assemblies [32], progressing into neuronal degeneration [36–39] and memory loss [40, 41]. A pure impairment of memory is how AD usually exhibits itself in its earliest clinical phase [36].

### Artificial plaque deposition

Scientists working on AD need to understand the pathogenic effects of Aβ better. While animal and in vitro models of Aβ exist and are most commonly used for this purpose [42], optogenetic methods of artificial plaque induction have also been introduced in recent years. Lim et al. [43] developed fluorescently labeled, optogenetically activated Αβ peptides that can oligomerize in vitro upon illumination. The authors concentrated on the question of how intracellular Aβ oligomers underlie the pathologies of AD and were able to put a distinction between the metabolic and physical damage of Aβ, and between the damage caused by light-induced Aβ oligomerization from mere Aβ expression. The physical damage caused by Aβ oligomers signifies tissue loss, a hallmark of late AD. Kaur et al. [44] went further and enhanced a similar method to generate in vivo Αβ aggregation.

### Dissecting hippocampal memory pathways

**Fig. 1:**
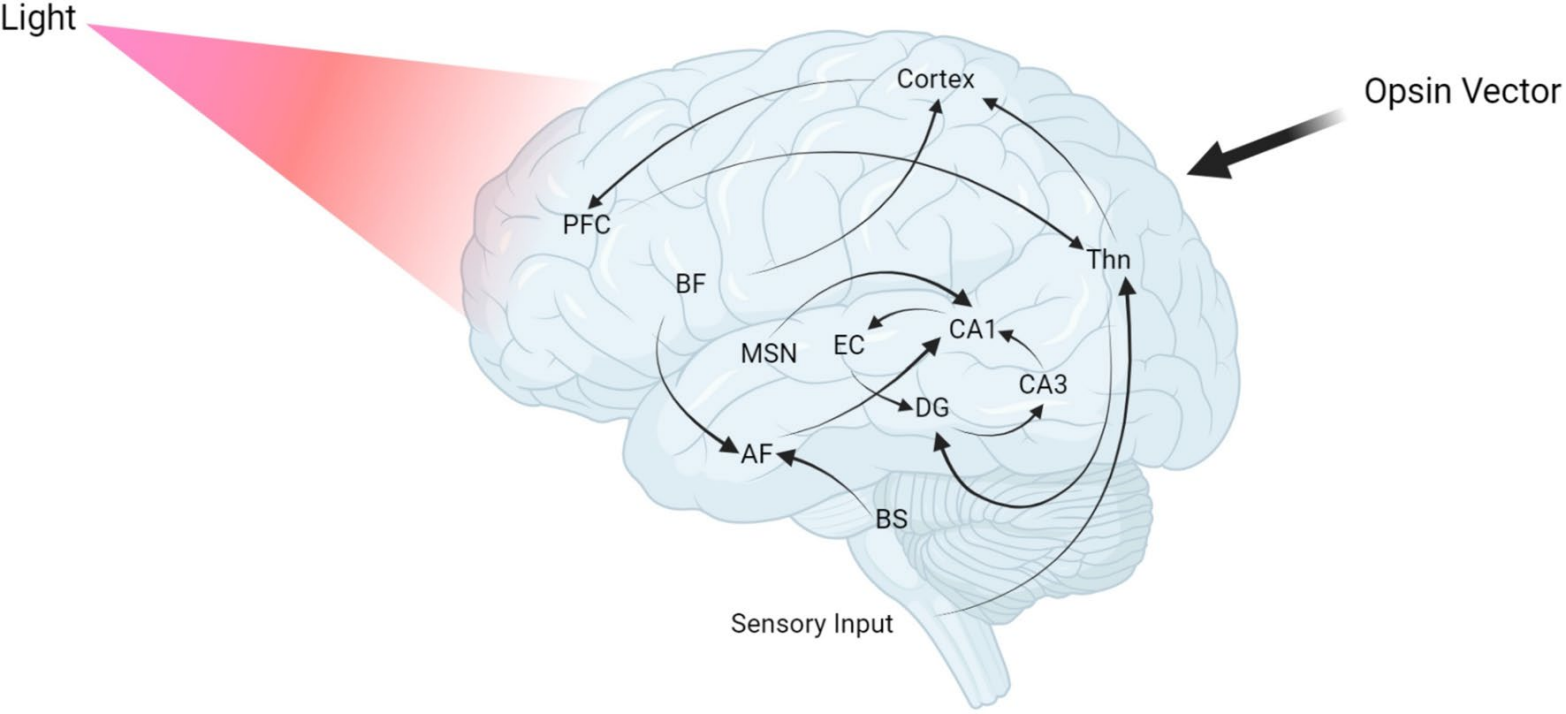
Optogenetics illuminates memory circuits impaired in AD

Memory impairment in the early stages of AD is limited chiefly to episodic memory, in which the hippocampus plays a crucial part [36] (Fig. 1). AD and similar memory disorders associated with this part of the brain have sparked interest in the hippocampus’s role in memory, and neurodegenerative memory loss research has extensively focused on pathology in the medial temporal lobe, primarily in the hippocampus [45]. The neural circuits in the dentate gyrus (DG) and the CA1 regions in the hippocampus, the first to receive signals from the cortex and the last to process them, respectively [46], were the earlier interests of scientists in the field.

It was established that optogenetic inhibition of pyramidal cells (PCs) in dorsal CA1 [47] or disinhibition through somatostatin-positive (SST) cells [48] reversibly disrupts memory acquisition. Inhibition of CA1 during recall also disrupts recall [47]. These findings underscored the importance of CA1 and suggested that a precise CA1 function is needed for both memory acquisition and recall.

Light-induced inhibition of dorsal DG, on the other hand, was found to impair only memory acquisition but not recall, whereas its hyperactivation [49] or disinhibition through hilar GABAergic neurons [50] impairs recall but not acquisition. However, this inhibition causes remarkable cognitive impairment when the mouse model needs to encode a new, conflicting memory rapidly. Consistent with former knowledge, these data revealed that CA1 is the terminal region from which the hippocampus emits output signals and is crucial for memory recall. In contrast, DG could be bypassed through alternative circuits, namely the intrahippocampal associative network of CA3 and the entorhinal cortex (EC) [51, 52].

The vast diversity of GABAergic interneurons (INs) in the hippocampus arms its microcircuits with the required flexibility for memory encoding and retrieval. Using optogenetics, it was found that a population of hippocampal GABAergic INs, known as oriens lacunosum-moleculare (OLM) cells, gatekeep the information flow in CA1, enabling the intrahippocampal transmission of information from the CA3 while reducing the influence of extrahippocampal inputs from the EC. These cells are interconnected and receive cholinergic inputs from different regions of the brain, suggesting that acetylcholine, acting through OLM cells, can control the mnemonic processes executed by the hippocampus [53].

Curious laterality in memory function is also observed in the hippocampus. Optogenetically silencing CA3 PCs on the left but not the right hippocampus impairs associative spatial long-term memory [54]. Aβ also selectively disrupts synaptic activity and facilitates long-term depression (LTD, see next section) in the left hippocampus [55], providing insights for further studies to understand the development of unusual brain lateralization in AD.

### Hippocampal rhythmic oscillations

Rhythmic activity in the hippocampus occurs within distinct frequency bands, namely theta (4–12 Hz) and gamma (30–120 Hz) oscillations. Slow (30–60 Hz) and fast (60–120 Hz) gamma oscillations have also been proposed recently to have functionally distinct roles in memory [56]. These oscillations are prominent in spatial memory processing [57] and synaptic plasticity [58, 59]. Two critical models of synaptic memory learning, long-term potentiation and depression (LTP and LTD), are also closely related to the hippocampal oscillations and are involved in learning and memory formation [60, 61]. Background abnormal accumulation of amyloid oligomers is believed to be the mechanism that causes dysfunction in hippocampal parvalbumin-positive (PV) and SST IN circuits [62], which are critically involved in the induction of these oscillations, and impairs LTP by extension [34, 63–65].

Indeed, exploiting optogenetics, it was found that AβO_1–42_ causes specific dysfunction in CA1 PC-to-PV IN and PV-to-PC synapses. Having no effect on PC-to-SST or SST-to-PC synapses, AβO selectively disrupts SST IN-mediated disinhibition to CA1 PC and impairs theta-nested gamma oscillation-induced spike-timing-dependent LTP [66]. AβO also increases the probability of the initial GABA release that depresses SST/PV IN's inhibitory input to CA1 PC selectively at theta and gamma frequencies, respectively [62]. These oscillations are reversible through optogenetic activation of neurons [66]. Light activation of SST and PV INs similarly restores AβO-induced theta and gamma oscillation peak power reduction, resynchronizing spike phases relative to the respective oscillations, and resynchronizes CA1 PC spikes [62].

Since theta frequency rhythmic oscillatory activity underlies a mechanism that can synchronize neural network activity during mnemonic processes [67], researchers commonly use theta-burst stimulation in order to induce LTP, especially in the hippocampus [68]. For instance, Yang et al. showed that selective optogenetic induction at theta-burst frequency in the pathway between EC layer II PCs and CA1 PV GABAergic neurons, a pathway that exhibits degeneration in AD, before the onset of symptoms, rescues degeneration of synapses and improves learning and memory function [69].

The CA1 is a point of convergence that receives gamma frequency inputs from upstream regions (CA3 and medial EC) and generates itself a faster [70] gamma oscillation. Butler et al. produced an optogenetic model of intrinsic CA1 gamma oscillations and showed that sinusoidal optical stimulation of CA1 at theta induces strong theta-nested gamma oscillations similar to in vivo, suggesting there exists a single gamma rhythm generator [71]. Using optogenetically induced theta–gamma oscillations, the authors later found that CA3 stimulation induces slower gamma oscillations in CA1 than stimulation of either medial EC or CA1 itself, where gamma oscillations are of similar frequency [72]. Optogenetic inhibition of PCs in CA1 also was not found to affect the power of the oscillations [70]. Altogether, these results reveal an excitatory-inhibitory feedback loop that underlies gamma oscillation generation in all three regions.

Increasing data is suggesting that brain network alterations rather than protein deposition could account for the early pathogenesis of AD and might even precede the canonical Aβ deposition [73], most notably the theta-gamma cross-frequency coupling [74]. Mondragon-Rodriguez et al. studied young mouse models of AD and found that not the PC inhibition by local INs nor the PV excitation by PCs, but the intrinsic excitability of PV cells was reduced in this condition. This impairment of cross-frequency coupling was not rescued by optogenetic activation of PV INs, which directly drive theta oscillations in the hippocampus, suggesting this damage does not directly result from an alteration of the underlying theta rhythm [75].

These hippocampal PV INs in the hippocampus are directly regulated by extrahippocampal networks as well, for example, medial septal PV cells (MSPVs) [76], which constitute a septohippocampal feedforward inhibitory control [77]. Optogenetic stimulation of MSPV projections in the hippocampus is associated with direct, frequency-specific pacing of hippocampal oscillations [78, 79]. In an important study, Etter et al. demonstrated how optogenetic gamma stimulation could activate MSPVs, thereby restoring hippocampal slow gamma oscillations amplitude and phase-amplitude coupling, and finally retrieving lost spatial memory despite significant plaque deposition [80].

### Corticohippocampal relations

Mounting evidence suggests that the prefrontal cortex (PFC) is preferentially vulnerable to AD-related pathology [81], possibly due to metabolic demands of this region [82]. For instance, the medial PFC is among the first brain regions to develop Aβ plaques [83]. This renders the PFC an important region to study for AD researchers.

It is known that a joint contribution of the medial PFC and hippocampus supports successful spatial working memory in rodents [84, 85]. Spellman et al. established that optogenetic disruption of the gamma synchrony between these regions impaired encoding, but not maintenance or retrieval, of spatial cues [86]. Optogenetic inhibition of excitatory medial PFC neurons similarly inhibited the activation of the entorhinal-hippocampal circuit and, therefore, impaired long-term associative memory formation [87]. Altogether these data reveal that encoding of long-term episodic memory is related to an early remodeling of circuits within the neocortex and that the PFC is a crucial regulator of hippocampal activation during encoding and long-term memory formation.

Cassel et al. reported that optogenetic stimulation of the reuniens-rhomboid nuclei (ReRh) of the thalamus induces LTP in CA1 and alters functions that are also sensitive to lesions of the hippocampus and the medial PFC [88]. On the other hand, Ito et al. reported that optogenetic silencing of Re reduced spatial memory-dependent CA1 activity, further establishing that projections from medial PFC via the Re to the hippocampus are crucial for spatial memory [89]. Maisson et al. similarly demonstrated that light-induced Re inhibition selectively disrupts memory encoding [90]. These studies suggest that thalamic nuclei contribute to the encoding of spatial information during working memory and may act as a gateway, relaying cortical input to the hippocampus.

The DG is the primary gate of the hippocampus and controls information flow from the cortex [91, 92], and granule cells (GCs) are the principal neurons in the DG which receive inhibition from both PV and SST GABAergic inhibitory INs. Lee et al. utilized cell-specific optogenetic perturbation and found that PV and not SST INs suppress GC responses among these cells. PV INs also control the onset, whereas SST INs regulate the later spikes [93].

The EC is another hotspot that regulates hippocampal activity. Mild cognitive impairment, which often precedes AD, is characterized by a significant neuronal loss in the EC, most notably in the layer II PCs [94, 95]. Bott et al. reported that a partial EC lesion generates hyperactivity in the DG, which could be abolished by optogenetic stimulation of hippocampal cholinergic fibers. These cholinergic fibers sprout in response to the lesion; therefore, control of DG hyperactivity by cholinergic sprouting seems to be involved in functional compensation of reduced EC glutamatergic input after a lesion of the EC [96].

### Slow waves and sleep

Slow oscillations are crucial for memory consolidation during sleep, the power of which is commonly diminished in AD. AD-associated sleep–wake cycle disruptions include disruptions in NREM slow-wave sleep. AD patients spend less time in NREM sleep and exhibit decreased slow-wave activity. Consistent with the critical role of SWS in memory consolidation reduced SWA is associated with impaired memory consolidation [97].

Kastanenka et al. reported that inhibitory activity within the cortical circuit is responsible for slow oscillation dysfunction since light activation of excitatory cortical neurons restored slow oscillations by synchronizing neuronal activity. While optogenetic driving of slow oscillation activity halted plaque deposition and prevented calcium overload associated with this pathology [98], the authors later found that attempting to propel this circuit at an increased rate yields opposite results [99], possibly due to activity-dependent acceleration of amyloid production. This phenomenon has also been demonstrated before, through optogenetic activation of the hippocampus [100] and is consistent with former knowledge that neuronal activation increases Aβ release from presynaptic terminals [101]. There is a positive feedback loop between amyloid/tau pathology and slow-wave activity (SWA) disruptions in AD that cause even further accumulations of amyloid and tau, possibly hinting at the utility of SWA disruptions for early AD diagnosis [97].

### The forebrain

The basal forebrain (BF) is another major controller of cortical and hippocampal activity, and its dysfunction coupled with a significant loss of its cholinergic neurons is observed in AD. Many of the BF neurons involved in said processes are, in fact, GABAergic, including a subpopulation of PV projection neurons [102]. Optogenetic stimulation of the cholinergic neurons activates the cortically projecting BF PV GABAergic neurons (CPBPGs), suggesting that the loss of cholinergic neurons in AD may partially impair cortical activation through CPBPGs [103].

CPBPGs seem to regulate gamma-band oscillations (GBO, 30–80 Hz, typically ~ 40 Hz) in the cortex. These oscillations are involved in higher cognitive functions such as attention and working memory, and their impairments are a feature of many disorders associated with dysfunction of cortical fast-spiking PV INs, including AD [104]. Indeed, Kim et al. showed that optogenetic activation or inhibition of CPBPGs can preferentially increase cortical GBO or reduce the ability of the cortex to generate GBO, respectively, indicating that this presumptively inhibitory input likely synchronizes cortical PV INs [105]. Optogenetic activation of BF PV neurons, however, unlike what is observed in hippocampal PV neurons, increases amyloid burden, suggesting that the beneficial effects of GBO on AD pathology depend on the method it is induced. In other words, activating BF inhibitory neurons preferentially suppresses cortical PV neurons rather than activating them [106]. Optogenetic stimulation of BF nuclei has also been used to establish BF modulation of sensory responses in the cortex [107].

Developing an optogenetic method for GBO induction, gamma entrainment using sensory stimuli (GENUS), Iaccarino, Singer et al. found that driving fast-spiking PV INs at gamma reduces levels of Aβ isoforms; hinting to a possible role of gamma rhythms in recruiting neuronal and glial responses to ameliorate AD pathology [108]. The main advantage of this study, was that this multi-sensory stimulation recruits multiple brain regions with a subsequent wider effect on the brain. It was later found that auditory GENUS boosts hippocampal function and affects microglia, astrocytes, and vasculature in the auditory cortex and hippocampus. Both auditory and visual GENUS can induce microglia clustering around plaques, which could explain how GBO induction reduces amyloid and tau burden throughout the neocortex [109].

### The cholinergic system

Brain cholinergic neurons are critical for memory function, and their loss contributes to memory impairment in AD. One role of these neurons is to elicit theta rhythm in the hippocampus during periods of learning when hippocampal synapses are in a state of heightened plasticity [58].

It has been established that optogenetic septal cholinergic input induces different types of hippocampal plasticity depending on the timing, all which are disrupted by Aβ exposure [110], impairs spatial memory formation when activated at goal location but not during navigation, reduces sharp-wave ripple incidence at goal location, and enhances theta-gamma oscillations during sleep [111]. These results highlight the importance of proper timing of cholinergic input in long-term memory formation, which could explain the relatively limited success of cholinesterase inhibitors in treating AD.

Betterton et al. found that acetylcholine release in CA3 enhances light-induced gamma oscillation power in lower and decreases it in higher concentrations, acting primarily through muscarinic receptors [112]. Aitta-aho et al. similarly found that while brainstem acetylcholine neurons transiently excite amygdala neurons through glutamate, BF acetylcholine neurons cause biphasic inhibition-excitation responses that synchronize amygdala activity. The authors suggested that the brainstem and the BF inputs to the amygdala might drive opposing learning behaviors [113].

Forebrain cholinergic neurons also regulate innate immune responses and inflammation, which is interesting to note, considering that anti-inflammatory regulation is often impaired in diseases associated with cholinergic dysfunction, including AD. In one study, selective optogenetic stimulation of BF cholinergic neurons was found to significantly reduce serum TNF levels, a marker of inflammation [114].

### Memory engrams

Hippocampal cells, thought to represent "memory engrams," have been optogenetically targeted to better understand the circuits involved in AD-related memory pathology. In a critical study, Roy, Arons et al. showed that in early AD amnesia, direct optogenetic activation of memory engram cells could retrieve lost memory, suggesting a retrieval rather than a storage impairment. The authors optically induced LTP at perforant path synapses of DG engram cells and reported restoration of both spine density and long-term memory, a reduction in which is the underlying mechanism of age-dependent amnesia that precedes plaque deposition [115]. Bostancıklıoğlu similarly showed that optogenetic manipulation of serotonin nuclei retrieve the lost memory by closing potassium channels on the memory engram cells, raising questions about the effects of serotonin on memory engram cells and pointing to the possible interface between the amyloid‐centric hypothesis of AD and the memory engram hypothesis in order to explain memory loss in AD [116].

In order to visualize memory traces, Denny et al. created a transgenic line of mice that allowed for the comparison between cells activated during encoding and expression of memory. Mice re-exposed to a context had more reactivated cells in the DG and CA3 than mice exposed to a novel context. Over time, these differences disappeared, in keeping with the observation that memories become generalized. Optogenetically silencing DG or CA3 cells that were recruited during encoding of a fear-inducing context prevented the expression of the corresponding memory. Mice with reduced neurogenesis displayed less contextual memory and less reactivation in the CA3, but surprisingly, regular reactivation in the DG [117]. The results suggest that distinct memory traces are located in the DG and CA3, but the strength of the memory is related to reactivation in the CA3.

### Emotions and memory

Depression is commonly observed in patients with dementia [118], such as AD [119]. Animal studies have also confirmed a causal relation in that depression can impair memory [120, 121]. The basolateral amygdala (BL) is famously being associated with emotion and motivation, playing an essential role in processing emotion-associated events. BL has neuronal fibers directly projecting to the hippocampus [122] and regulates long-term potentiation of the dorsal hippocampus [123] and hippocampal plasticity [124].

Yang et al. developed mouse models of learned hopelessness and learned hopefulness (LHL and LHF), which are models of memory impairment and enhancement resulting from negative and positive emotions, respectively. The authors found that opposite scaling of the excitatory monosynaptic connection between posterior BL (BLP) and ventral CA1 governs the modified spatial learning and memory. More interestingly, optogenetic disruption of this circuit abolishes the effects of LHF and impairs synaptic plasticity, whereas its stimulation rescues the LHL-induced memory deficits [125].

“Sundowning” is another emotional disturbance in AD, characterized by early-evening agitation and aggression [126]. Exploiting optogenetic mapping technics, Todd et al. found that a population of GABAergic subparaventricular zone neurons, which are major postsynaptic targets of the central circadian clock, receive input from neurons in the aggression regulating region of the hypothalamus and revealed a functional circuit by which the circadian clock regulates aggression [127], explaining the daily rhythmicity of this phenomenon.

### Neurogenesis

The hippocampus, most notably the DG, is a plastic and vulnerable structure in which neurogenesis occurs in embryonic and postnatal periods, and impairments in neurogenesis are one of the key manifestations of Aβ pathology [128]. Morgun et al. found that aberrant mechanisms of development of stem and progenitor cells caused by Aβ_1-42_ can be partially restored through targeted optogenetic activation of certain astrocytes in the neurogenic niche [129].

Duan et al. have recently presented an optogenetic tool to study the nerve growth factor/tropomyosin receptor kinase A signaling pathway, which plays a key role in neuronal development, function, survival, and growth, and is implicated in neurodegenerative disorders. However, this tool has yet to be used in the context of AD [130].

### Drug function

Optogenetic tools have also been used to test drug function. Memantine has been shown to improve cognitive functions AD models. Using optogenetics, it was established that memantine enhanced EC to CA1 synaptic neurotransmission and promoted dendritic spine regeneration of EC neurons that projected to CA1 [131]. Caffeine consumption prevents memory deficits in aging and AD through the antagonism of adenosine receptors, optogenetic activation of which in the hippocampus impairs spatial memory performance [132].

### Neurovascular dysfunction

AD is associated with neurovascular dysfunction, pericyte loss, and reduced cerebral blood flow [133]. Optogenetic manipulation of the activity of individual and small clusters of mural cells and consequent imaging techniques together with system modeling methods [134] have been used to allow the investigation of pericyte and smooth muscle cell physiology and their role in regulating cerebral blood flow [135]. One study, for instance, noted that optogenetic excitation of pericytes results in contraction followed by constriction of the underlying capillary leading to a decrease in capillary diameter and reduced capillary RBC flow [136].

### Cellular stress

DNA damage is intimately connected to aging and the manifestation of age-related neurodegenerative disorders such as AD [137]. Suberbielle et al. showed that DNA double-strand breaks that naturally occur during exploration of a new environment are increased in AD and are even more severe after optogenetic stimulation. The authors found that suppressing aberrant neuronal activity, and improving learning and memory, normalized DSB levels [138].

Αβ_1–42_ induces oxidative stress in AD [139]. Using zebrafish as a model, Formella et al. utilized a genetically encoded photosensitizer that produces reactive oxygen species upon stimulation to study oxidative stress and neurodegeneration. It was found that neural cells undergo stress and cell death similar to what is seen in several neurodegenerative diseases, including AD [140].

## Role of optogenetics in Alzheimer's disease treatment

### Conventional AD treatments

AD treatment remains symptomatic without changing the disease prognosis, and there is no definitive cure available for it, which could slow the disease progression and mitigate cognitive and memory impairments [141, 142]. To date, only seven medical treatments have been approved for AD by the United States Food and Drug Administration (FDA): Six treatments (including Donepezil, Galantamine, Rivastigmine, Memantine, Memantine + Donepezil, Suvorexant) act to control symptoms rather than changing the course of the disease, and one treatment (Aducanumab) may delay the clinical decline [143]. Most clinical therapeutic approaches use passive immunotherapies with monoclonal antibodies to clear Aβ peptides and tau proteins [144].

The recent approval of Aducanumab reignited the interest around novel therapeutic approaches, as it marks the first approved disease-modifying therapy for AD [145, 146]. Lamentably, Aducanumab is one of the very few clinical trials that have investigated AD treatments in the last two decades. The general outcome for these trials is considered a big failure, with an overall success rate of 0.4% during the 2002–2012 period [147]. This rate emphasizes the need to identify different therapeutic plans for AD treatment.

### Optogenetics and AD treatment in animal studies

**Fig. 2:**
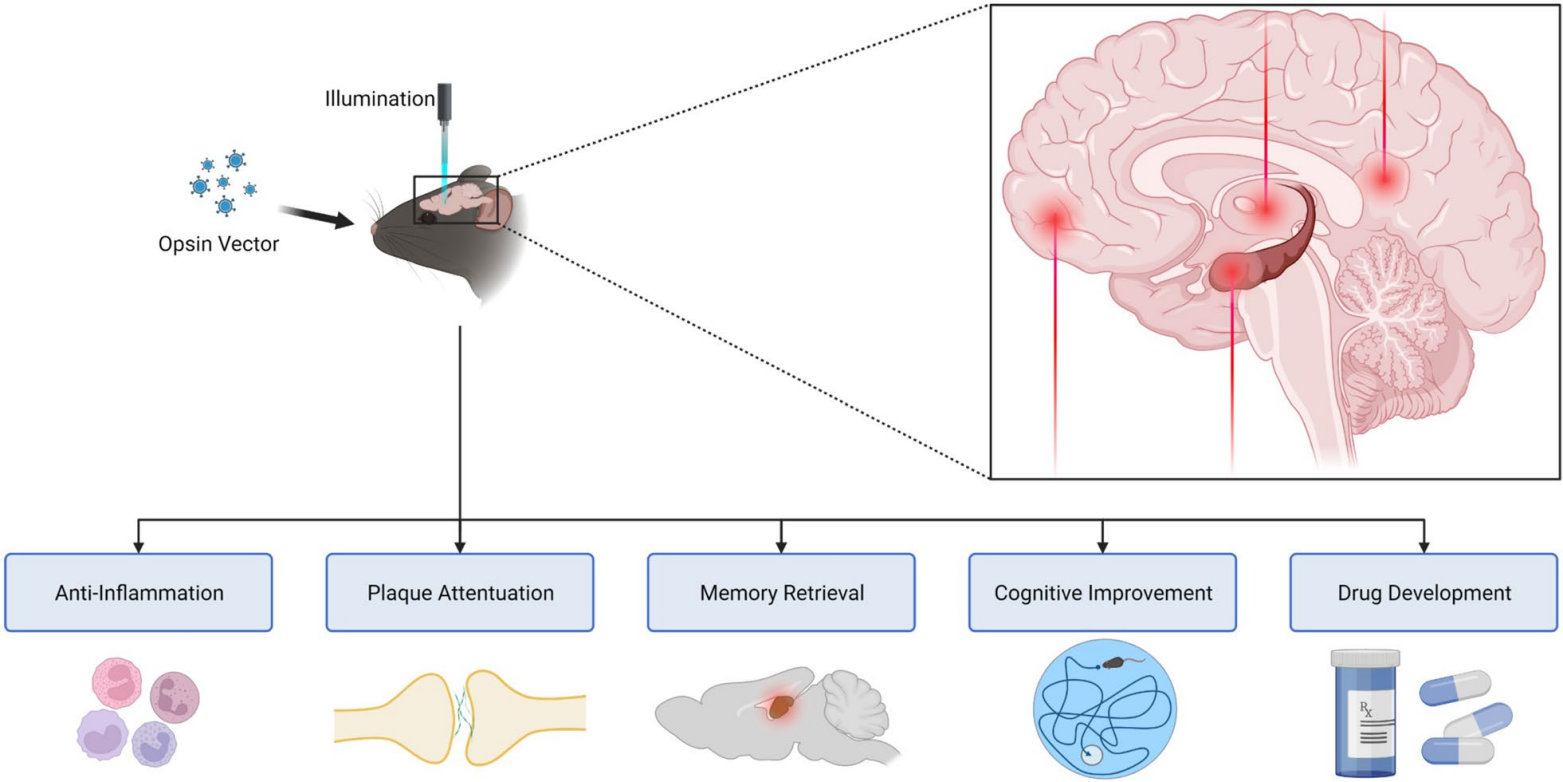
Optogenetics ameliorates AD pathology, augments pharmacological studies, and points to valuable spots for future interventions

Optogenetics could, in theory, be an alternative therapeutic strategy against AD. However, as the field stands today, there are major translational obstacles to overcome (see section ‘Clinical application challenges’) if optogenetic methods are to be embedded into the clinic. These tactics can embark on better therapeutic interventions, alongside their other advantages, for pathophysiological studies and screening purposes [2]. The use of optogenetics, even if not directly therapeutic, can also provide insight into the mechanism of action and development of other forms of treatment [148] (Fig. 2). The chief advantage of using optogenetics over conventional electrical or pharmacological techniques may be more precise targeting of specific neural elements, greater cellular and temporospatial specificity, and reduced off-target effects [149–151].

Different murine studies on AD model mice have shown promising results. For example, Yang et al. found an effective way for rescuing synaptic decay and developing spatial learning and memory by optogenetic activation of EC layer II-CA1 PV synapses with theta-burst stimulation (see section ‘hippocampal rhythmic oscillations’) [69]. Roy et al. suggested that the dendritic spine density loss in DG neurons can be reversed in early AD stages using optogenetic techniques. They also reported that stimulating photo-activating DG engram cells could lead to episodic memory recall [115] (see section ‘Memory engrams’). Perusini et al. separately studied optogenetic stimulation of DG and reported reactivation of previously learned neural memory ensembles and memory improvement in AD model mice [152].

Gamma oscillations (see section ‘Hippocampal rhythmic oscillations’) are essential for higher cognitive functions and sensory responses [153–155], and changes in them (20–50 Hz) have been observed in various neurological conditions, including AD [156, 157]. Iaccarino, Singer et al. used optogenetics to spike these oscillations at 40 Hz in the hippocampus of 5XFAD mice and reported a dramatic reduction of Aβ peptides followed by microglia response [108] (see section ‘The forebrain’).

Robinson et al. investigated the effect of optogenetic techniques on neurotransmitter signals and ascertained that optogenetically activating glutamatergic neurons could facilitate learning and memory by theta-wave generation in the hippocampus [158]. Optogenetic activation of glutamatergic neurons in the bilateral DG in AD has been shown to improve working and short-term but not long-term memory, associated with increased expression of glutamate receptors in the hippocampus [159, 160]. It was also established that glutamate receptor upregulation varies in various hippocampus regions, suggesting that a single-target optogenetics strategy has spatial limitations and a multiple targeted optogenetics approach to AD therapy should be explored [160].

GABA levels are different in wild and AD model mice brains. GABA downregulates Αβ uptake in neurons; therefore, relatively high levels of GABA decrease Αβ-induced cytotoxicity. GABA treatment also decreases basal levels of cell death. Application of GABA during early life at an early age but not at older ages can improve cognitive function significantly. Activating or suppressing GABA(A) receptors by optogenetic methods also confirmed that GABA activation at young age ameliorated Aβ pathology, suggesting early life GABA as AD treatment [161]. In another research, Zhang et al. found that optogenetic stimulation of GABAergic neurons in the hippocampus of APP/PS1 mice induced autophagy, mitigated neuroinflammation, reduced Aβ fragments, and completely reversed the learning impairment [162].

Mancuso et al. suggested that despite the anatomical depth of their cell bodies, cholinergic projection neurons provide a better target for systems-level optogenetic modulation in AD treatment than cholinergic INs found in several brain regions, including the cortex and the striatum that are activated in traditional deep brain stimulation [163].

### Feasible brain sites for optogenetic-mediated therapy

Although the whole brain structure acts as a unit and the activity of the whole system should be considered for interventional studies on AD and memory loss, it is not always practical to modify the entire brain to access the effect of optogenetic stimulation [45], especially given that most vectors modify the genome in order to reach stable levels of opsin expression. Some studies have identified hippocampal formation as the most prominent site for interventions to enhance memory [164–166], together with sites that work in conjunction with the hippocampus, such as frontal and retrosplenial cortices [167–170]. Other studies suggested targeting major input/output regions such as the EC and area CA1 [69, 171–173].

Today, the main focus is on small brain structures which innervate and regulate hippocampal and cortical regions. One of these structures is the small thalamic nuclei [174–176], particularly anterior thalamic nuclei (ATN). ATN has a strategic position and extensive cortical connections [177–179], and its anteroventral nucleus (AV) comprises a large population of theta frequency cells that coordinate memory [180]. Another small structure is the mammillary body (MB) which neurons are connected to ATN and fire at theta frequency, but its location on the ventral surface of the brain makes it a difficult target for optogenetics [181]. The nucleus Re is the third site of interest because of its dense reciprocal connections with the PFC and the hippocampus, directly innervating the CA1 area [175]. The Re connections suggest it has a vital role in mnemonic functions [182, 183]. One specific study reported working memory impairment due to delta frequency optogenetic stimulation of transduced Re terminals [184].

### Clinical application challenges

The main hurdle is taking research results from the laboratory into the clinic, specifically the delivery of optogenetic tools in patients. As Shen et al. stated, the minimum requirements of an ideal clinical optogenetic therapy would be: (1) a safe and efficient gene delivery vehicle; (2) targeting of the gene delivery vehicle to the tissue of interest; (3) a delivery vehicle, transgene, and therapeutic protein gene-product, that is non-immunogenic and non-mutagenic; and (4) an optogenetic protein that is highly sensitive to light in the red to near-infrared wavelength range (to keep light doses low, maximize light penetration, and minimize photodamage) [185].

#### Gene delivery vehicle

Presently, viral vector-based transduction is the most forward-looking, persuasive, and commonly used method to deliver foreign genes to specific tissues in mammals [186]. Different phases of clinical trials also proved the safety of using the adeno-associated virus (AAV) as a viral agent in humans [187].

#### Targeting the gene to a specific tissue

Local virus injections are used to deliver optogenetic tools to the central and peripheral nervous systems in rodents. This prevents the virus from direct contact with the bloodstream and its circulating antibodies. However, local stereotaxic injection into the human brain is not as easy as rodents and will require imaging and expert analysis. Engineered AAV capsids such as the AAVDJ are found to have increased spreading capacity with promising perspectives [188].

Other alternatives to this approach include delivering AAV to the cerebrospinal fluid, which transfers the gene throughout the brain and spinal cord [189]. Nevertheless, the tight junctions of ependymal cells will restrict AAV entry into the brain parenchyma and makes its use less practical, unlike rodents [190].

#### Light delivery

Delivering light to the brain is also more complicated in primates and humans than rodents because of the larger size of their brains, and the deeper positioning as some of the important target structures such as the hippocampus and the thalamus (see section ‘Feasible brain sites for optogenetic-mediated therapy’). Strategies such as using an epidural optic fiber or small-scale bio-optoelectronic implants to deliver light are considered invasive procedures and may be helpful in mice. However, it is far from being used in humans and may be accompanied by side effects, such as tissue overheating and damage by light, tissue scarring, or infection [191, 192]. This makes light delivery possibly the largest obstacle on the path of therapeutic optogenetics.

#### Immunogenicity and genotoxicity

All gene- and protein-based therapies, including optogenetic-based therapies, bring the possibility of adverse immune responses. So, the potential risk of opsin, a foreign protein antigen, as an autoimmune agent in patients must be considered if safe optogenetic therapeutics are to be developed in the future [193]. Moreover, it must be taken into account that there may be pre-existing immunity against the AAV delivery vehicle itself. Even at low levels, anti-AAV antibodies can prevent the vehicle from reaching its destination [194, 195].

### Recent therapeutic developments

Despite all the challenges, optogenetics has been safely and effectively applied to awake non-human primate rhesus macaques (*Macaca mulatta*) [196, 197], which is remarkable, as primate studies take the field closer to the clinic. Another impressive effort was the first reported case of partial functional recovery in a neurodegenerative disease after optogenetic therapy, reported in July 2021. In this effort, the partial vision was recovered in one participant, a 58-year-old male diagnosed with retinitis pigmentosa [198]. This, despite the challenges listed above, shows a promising prospective for considering optogenetics as an alternative in the future therapeutic methods of neurodegenerative conditions, including AD.

## Data Availability

Not applicable.

## Abbreviations

AD: Alzheimer's disease
IN: Interneuron
LFP: Local field potentials
LTP: Long term potentiation
SST: Somatotropin-positive
PV: Parvalbumin-positive
PC: Pyramidal cell
EC: Entorhinal cortex
PFC: Prefrontal cortex
DG: Dentate gyrus
BF: Basal forebrain
Aβ: Amyloid beta
AβO_1–42_: Amyloid beta (1–42) oligomers
GC: Granule cells
